# Parallel screening of FDA-approved antineoplastic drugs for identifying sensitizers of TRAIL-induced apoptosis in cancer cells

**DOI:** 10.1186/1471-2407-11-470

**Published:** 2011-11-01

**Authors:** David J Taylor, Christine E Parsons, Haiyong Han, Arul Jayaraman, Kaushal Rege

**Affiliations:** 1 Chemical Engineering, Arizona State University, 501 E. Tyler Mall, ECG 303, Tempe, AZ 85287-6106, USA; 2 The Translational Genomics Research Institute (TGen), 445 N. Fifth Street, Phoenix, AZ 85004, USA; 3 Artie McFerrin Department of Chemical Engineering, 3122 TAMU, Texas A&M University, College Station, TX 77843, USA

## Abstract

**Background:**

Tumor Necrosis Factor-α Related Apoptosis Inducing Ligand (TRAIL) and agonistic antibodies to death receptor 4 and 5 are promising candidates for cancer therapy due to their ability to induce apoptosis selectively in a variety of human cancer cells, while demonstrating little cytotoxicity in normal cells. Although TRAIL and agonistic antibodies to DR4 and DR5 are considered safe and promising candidates in cancer therapy, many malignant cells are resistant to DR-mediated, TRAIL-induced apoptosis. In the current work, we screened a small library of fifty-five FDA and foreign-approved anti-neoplastic drugs in order to identify candidates that sensitized resistant prostate and pancreatic cancer cells to TRAIL-induced apoptosis.

**Methods:**

FDA-approved drugs were screened for their ability to sensitize TRAIL resistant prostate cancer cells to TRAIL using an MTT assay for cell viability. Analysis of variance was used to identify drugs that exhibited synergy with TRAIL. Drugs demonstrating the highest synergy were selected as leads and tested in different prostate and pancreatic cancer cell lines, and one immortalized human pancreatic epithelial cell line. Sequential and simultaneous dosing modalities were investigated and the annexin V/propidium iodide assay, in concert with fluorescence microscopy, was employed to visualize cells undergoing apoptosis.

**Results:**

Fourteen drugs were identified as having synergy with TRAIL, including those whose TRAIL sensitization activities were previously unknown in either prostate or pancreatic cancer cells or both. Five leads were tested in additional cancer cell lines of which, doxorubicin, mitoxantrone, and mithramycin demonstrated synergy in all lines. In particular, mitoxantrone and mithramycin demonstrated significant synergy with TRAIL and led to reduction of cancer cell viability at concentrations lower than 1 μM. At these low concentrations, mitoxantrone demonstrated selectivity toward malignant cells over normal pancreatic epithelial cells.

**Conclusions:**

The identification of a number of FDA-approved drugs as TRAIL sensitizers can expand chemotherapeutic options for combination treatments in prostate and pancreatic cancer diseases.

## Background

Tumor Necrosis Factor-α Related Apoptosis Inducing Ligand (TRAIL) is a member of the Tumor Necrosis Factor (TNF) super-family of cytokines that engages the cellular apoptotic mechanism upon specific binding to death receptors (DRs) 4 and 5 on the cell surface [1]. TRAIL has attracted significant attention in recent years due to its ability to selectively induce apoptosis in transformed (malignant) cells while demonstrating little cytotoxicity in normal cells [2-7]. TRAIL binds cell-surface death receptors (DR4 and DR5) as a homotrimer and triggers the formation of the Death-Inducing Signaling Complex (DISC); the Fas-Associated Death Domain (FADD) and caspases 8 or 10 are recruited to the DISC from the cytoplasm. The proteolytic activation of initiator caspases leads to the subsequent activation of executioner caspases (e.g. caspase-3), which ultimately results in apoptosis in Type I Cells. Activation of caspase-8 engages the mitochondria-amplified apoptosis machinery in Type II cells [7]. The binding of TRAIL to decoy receptors (DcR) 1 and 2 has also been demonstrated; it is hypothesized that these receptors play a role in maintaining the homeostasis of TRAIL activity in vivo [2,8].

Recombinant TRAIL induces apoptosis in a variety of human cancer cell lines including those of breast, colon, lung, prostate, liver, leukemia, lymphoma, and neuroblastoma [4,6,8,9]. TRAIL has also demonstrated potent anti-tumor activity in a number of xenograft models including those of colon and breast carcinomas [10-12]. Soluble TRAIL variants are well tolerated in mice and chimpanzees [13] and demonstrate minimal cytotoxicity towards primary human hepatocytes and endothelial cells in culture [7,14]. As a consequence of the selectivity towards malignant cells, certain TRAIL formulations (e.g. non-histidine tagged TRAIL) are considered safe for potential therapeutic applications [15].

Although TRAIL and agonistic antibodies to death receptors 4 and 5 are promising candidates for cancer therapy, many tumor cells are inherently resistant or acquire resistance to TRAIL-mediated apoptosis. Commonly implicated resistance mechanisms include dysfunction of the Fas-Associated Death Domain (FADD)/improper assembly of the Death-Inducing Signaling Complex (DISC) [16], loss of caspase-8 activity [17-19], constitutively active Akt/protein kinase B [20], and over-expression of anti-apoptotic proteins such as c-Flip [16,21] and Bcl-2 [22]. As a result, therapeutic strategies involving DNA-damaging radiotherapy [23,24], genotoxins [25,26], and peptides [27] have been investigated for enhancing cancer cell sensitivity to TRAIL [25] and/or agonistic antibodies against DR4/DR5 [28].

Here, we report the parallel screening of fifty-five FDA-approved and foreign-approved chemotherapeutic drugs in order to identify existing anti-cancer drugs that might act as TRAIL sensitizers in resistant prostate and pancreatic cancer cells. Drugs were first pre-screened individually (single agent treatment) for toxicity at a concentration of 20 μM using TRAIL-resistant PC3-TR prostate cancer cells; candidates that resulted in greater than 70% reduction in cancer cell viability were screened for TRAIL sensitization activity at a lower concentration of 10 μM. A total of fourteen potential TRAIL sensitizer leads, including six whose TRAIL sensitization activities were previously unknown, were identified from the screen. Five leads were further characterized in prostate and pancreatic cancer cells.

## Methods

### Cell Culture

Two human prostate cancer cell lines (PC3, and PC3-TR), three human pancreatic cancer lines (Panc-1, MIAPaCa2, and BXPC-3) and one immortalized human pancreatic epithelial cell line (HPDE6) were used in the current study. PC3-TR (TR: TRAIL resistant) [29] cells were a generous gift from Dr. Aria Olumi at the Massachusetts General Hospital in Boston, MA. Cells were grown in 75 cm^2 ^Corning cell culture flasks with RPMI 1640 tissue culture media supplemented with 10% Fetal Bovine Serum and 1% penicillin/streptomycin (10000 units/mL penicillin G and 10000 μg/mL streptomycin) at 37°C with 5% CO_2_.

### Reagent/Drug Preparation

The Johns Hopkins Chemical Compound Library (JHCCL) [30] was purchased from The Johns Hopkins University School of Medicine. The library contains a total of 1514 FDA- and foreign-approved drugs. The anti-neoplastic plate (plate #1) consists of 55 FDA-approved approved anti-cancer drugs and was employed in the screen for identifying TRAIL sensitizers. All stock solutions of the drugs from the library were supplied at a concentration of 10 mM in either DMSO or water. For expanded dose-response experiments, additional doxorubicin and mitoxantrone were purchased from Sigma while gemcitabine, mithramycin, and thioTEPA were obtained through the NCI/NIH Developmental Therapeutic Program. TRAIL was purchased from R&D Systems and reconstituted in PBS at a 10 μg/mL stock concentration in 50 μL aliquots to prevent multiple freeze/thaw cycles. All solutions were prepared to ensure that the final solvent (DMSO or water) concentration in cell treatments would be less than 1% (v/v) to limit non-specific activity.

### Single Agent and Combination Treatments

Cells were plated in 96 well plates at a density of 8,400 cells/well and incubated at 37°C and 5% CO_2 _for approximately 24 hours. For single-agent treatments, cells were exposed to drug candidates at a concentration of 20 μM for 24 hours at which point, cell viability was determined using the MTT (3-(4,5-dimethylthiazolyl-2)-2,5-diphenyltetrazolium bromide) assay (described below). Single-agent TRAIL treatments were carried out similarly; a dose range of 0-100 ng/mL of TRAIL was used. For sequential combination treatments, cells were first treated with a sensitizer drug candidate for 24 hours. The media was then removed, replaced with fresh serum-containing media, and the cells were treated with TRAIL. Cells were incubated for an additional 24 h after which, viability measurements were carried out using the MTT assay. In order to determine if dosing of the combination treatment had an effect on the efficacy, simultaneous combination treatments were carried out by treating cells with the sensitizer drug and TRAIL at the same time for 24 h at which point, cancer cell viability was determined using the MTT assay.

### Determination of Cell Viability

Cell viability was assessed using the MTT cell proliferation assay (ATCC CA#30-1010k). Following addition of the MTT reagent (2 h at 37°C), cells were treated with a lysis buffer from the kit and kept at room temperature in the dark for two hours in order to carry out complete lysis and to solubilize the MTT product. The absorbance of each well was measured using a Biotech Synergy 2 Multi-Detection Microplate Reader at 570 nm. Each experiment included a set of blank wells (media only), a live control (no treatment) and a dead control (200 μL of 10 μM H_2_O_2 _or 1.5 μL of 20 μM Quillaja were employed for inducing death in the cell population). Background absorbance was measured using the blank and subtracted from all absorbance measurements. In the case of drugs that potentially interfered with the assay, a separate set of media-only wells were treated with equivalent volumes of the drug, and the measured absorbance was subtracted as the background. This was carried out to eliminate any bias caused by the natural absorbance of the drug itself. Fractional cell viability was calculated as: (OD of sample - OD of dead control)/(OD of live control - OD of dead control) where OD is the optical density. Percentage cell viability was calculated by multiplying the fractional viability by 100. Data are plotted as percentage reduction in cell viability compared to control (untreated cells) in which, a 0% value on the graph means 100% viability and 100% value on the graph means 0% viability.

### Statistical Analyses

Screening experiments were carried out in duplicate and expanded dose responses with identified leads were performed at least in triplicate. Data are presented as the mean ± one standard deviation. The standard deviation of each set was calculated based on the variation between experiments. ANOVA was performed using the t-test function in Microsoft Excel. Analysis of the single-agent treatment was performed in order to determine whether or not a drug had a significant effect when compared to the live (untreated) control. Efficacies of sensitization were determined by comparing the decrease in cell viability following combination treatments (i.e. drug in combination with TRAIL) to the reduction in cell viability as a consequence of the additive effect of single-agent treatments (drug alone + 10 ng/mL TRAIL alone).

### Live/Dead Analysis

As an alternate assessment of cell viability, a calcein AM/ethidium homodimer-1 (EthD-1) viability/cytotoxicity kit (Invitrogen L3224) was used to measure treatment efficacy for a select set of combination treatments. Briefly, a working solution of 2 μM calcien AM and 4 μM ethidium homodimer-1 (EthD-1) was prepared in a solution of sterile 1× PBS. The working solution was then added to each well of the cell culture plate and incubated at 37°C with 5% CO_2 _for 30-45 minutes. Fluorescence imaging was then carried out using a Zeiss Observer D1 fluorescent microscope. A 38 HE filter set (Excitation: 470/40; Emission: 525/50) was used to image the fluorescence of the calcein AM (green fluorescence) while a 43 HE filter set (Excitation: 550/25; Emission: 605/70) was used to measure the fluorescence of the EthD-1 (red fluorescence).

### Annexin V and Propidium Iodide Analysis

An annexin V/propidium iodide assay (Invitrogen L3224) was carried out to determine if combination treatments induced apoptosis in cells. Briefly, a working solution of 2% annexin V and 1 μg/mL propidium iodide (PI) was prepared in a solution of 1× annexin binding buffer. The working solution was added to each well of the cell culture plate and incubated at room temperature for 15-20 minutes. Fluorescence imaging was then performed using a Zeiss Observer D1 fluorescent microscope. A 38 HE filter set (Excitation: 470/40; Emission: 525/50) was used to measure the fluorescence of the annexin V (green fluorescence) while a 43 HE filter set (Excitation: 550/25; Emission: 605/70) was used to measure the fluorescence of the propidium iodide (red fluorescence).

### Image Analysis

All image processing was performed using ImageJ [31] image processing software. For the live/dead analysis the threshold of the fluorescent image was adjusted so that any background noise was removed and that the boundary between individual cells was well defined. The image was then converted to a binary format. In some cases, it was difficult to differentiate between cell boundaries in which case the Watershed process was used to distinguish between individual cells while ensuring that any cells that were incorrectly divided were accounted for [32]. The "Analyze Particles" function was then used to obtain a final cell count. The live cell count was then normalized against the live control cell count to give an indication of the cell viability. The brightness of the images was adjusted so that the background had zero pixel intensity value in case of annexin V/PI analyses. Next, false color was applied to the image; green was applied for the annexin V stains and red was applied to the PI stains.

## Results and discussion

In the current study, we screened a small library of fifty-five FDA- and foreign-approved antineoplastic drugs from the Johns Hopkins Clinical Compound Library (JHCCL) in order to identify chemotherapeutics that sensitize prostate and pancreatic cancer cells to TRAIL-induced apoptosis. Identification of FDA-approved drugs as TRAIL sensitizers is an attractive discovery strategy since it is possible to rapidly translate these novel combinations to the clinic. PC3-TR (TR: TRAIL resistant) human prostate cancer cells were used in the primary screening, since it was hypothesized that lead candidates discovered for this resistant cell line might be relevant to clinical phenotypes that develop resistance to TRAIL. The cell line demonstrated low susceptibility to single-agent TRAIL treatments; a 20% loss of viability reduction was observed in PC3-TR cells at concentrations as high as 100 ng/mL (Additional File 1). We employed a TRAIL concentration of 10 ng/mL in subsequent combination treatment experiments in order to keep the TRAIL dose at a minimum; under these conditions, single-agent TRAIL induced a loss of viability in approximately 4% of the PC3-TR cell population. To our knowledge, these are the first screening experiments carried out with the PC3-TR TRAIL-resistant prostate cancer cell line.

### Identification of FDA-Approved Drugs as TRAIL Sensitizers in Prostate and Pancreatic Cancer Cell Lines using Parallel Screening

**Figure 1:**
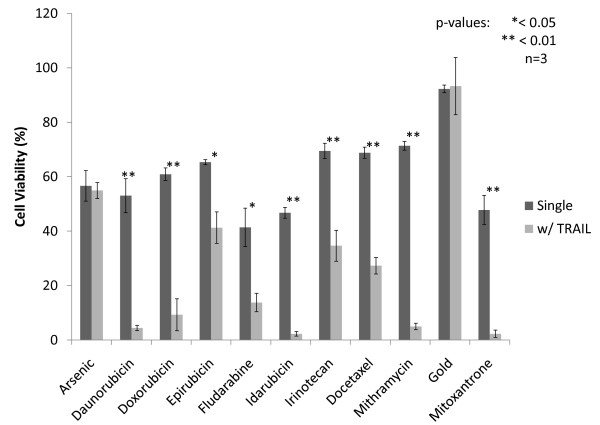
**Parallel screening of drugs from the Johns Hopkins Chemical Compound Library as TRAIL sensitizers**. PC3-TR human prostate cancer cells were first incubated with drug treatments (10 μM) for 24 hours after which time the media was removed, refreshed and cells were treated with TRAIL (10 ng/mL) for an additional 24 hrs.

**Figure 2:**
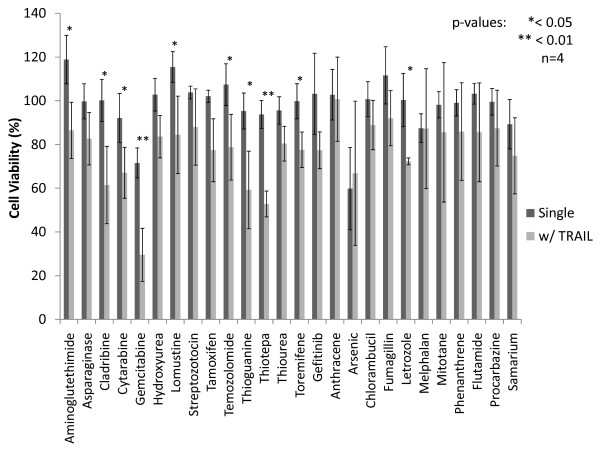
**Parallel screening of drugs from the Johns Hopkins Chemical Compound Library (JHCCL) as TRAIL sensitizers**. PC3-TR cells were first incubated with drug treatments (20 μM) for 24 hours after which time the media was removed, refreshed and cells were treated with TRAIL (10 ng/mL) for an additional 24 hrs.

The overall screening schematic is shown in Additional File 2. PC3-TR cells were initially treated with 20 μM of each drug in order to carry out an initial screen for drug toxicity. Eleven drugs (arsenic, daunorubicin, doxorubicin, epirubicin, fludarabine, idarubicin, irinotecan, docetaxel, mithramycin, gold, mitoxantrone) resulted in > 30% loss in PC3-TR cell viability at 20 μM in the initial toxicity screen (Additional File 3) and were screened for their TRAIL sensitization activities at a lower concentration of 10 μM (Figure 1). Use of lower drug concentrations (10 μM) limits the single agent toxicity, which can also allow for identification of synergistic interactions with TRAIL. Forty-four drugs from the library demonstrated cell viabilities higher than 70% at a concentration of 20 μM and were screened for their ability to sensitize PC3-TR cells to TRAIL under these conditions (Figure 2).

Combination treatments with sensitizer drugs (24 h) followed by TRAIL (24 h) were carried out in a sequential treatment format to identify candidates that sensitized PC3-TR cells to TRAIL-induced apoptosis. Combination treatments that induced statistically significant (p < 0.05) reduction in the viability of PC3-TR cells, compared to the sum of the individual treatments (i.e. additive effects of TRAIL alone + chemosensitizer drug alone), were identified as leads. Nine drugs, daunorubicn, doxorubicin, fludarabine, idarubicin, irinotecan, docetaxel, mithramycin, mitoxantrone and epirubicin, were identified as lead sensitizers of TRAIL-induced apoptosis from the 10 μM screen (Figure 1). Of these, seven have been previously identified as TRAIL sensitizers for different malignancies, but only five have been reported for prostate cancer (Table 1) [33-37]. To our knowledge, two drugs, idarubicin and mitoxantrone, have not been previously described as TRAIL sensitizers for any cancer cell type and are therefore new candidates that can be used in combination with TRAIL. The screen with 20 μM drug concentration identified the following drugs as TRAIL sensitizer leads: cladribine, cytarabine, gemcitabine, thioguanine and thioTEPA (Figure 2). Of these drugs, gemcitabine has been previously shown as a TRAIL sensitizer in different malignant cells, including prostate and pancreatic cancers [26]. Taken together, the two screens led to the identification of fourteen chemotherapeutic drug leads that sensitize PC3-TR prostate cancer cells to TRAIL-induced apoptosis; other candidates that demonstrated moderate efficacies (for example, toremifene, lomustine, temozolomide, aminoglutethimide, and letrozole) were also identified but not pursued further due to the presence of more promising leads. These results indicate that the screen accurately identified both, existing as well as new TRAIL sensitizers from the drug library. A summary of effective TRAIL sensitizers from the screen, along with their corresponding efficacies for reducing cancer cell viability, is provided in Table 1.

**Table 1:** Summary of drugs identified as TRAIL sensitizing agents and their previously known activity.

10 μM							
**Drug Name**	**Drug Alone**	**Drug Alone + TRAIL Alone** **(Additive)**	**Combination Treatment**	**p-Value**	**Known TRAIL Sensitizer?**	**Known TRAIL Sensitizer in Prostate Cancer?**	**Known TRAIL Sensitizer in Pancreatic Cancer?**

Daunorubicin	47	51.3	95.6	< 0.01	Yes	No	No
Docetaxel	31.2	35.5	72.7	< 0.01	Yes	Yes	No
**Doxorubicin**	**39**	**43.3**	**90.8**	**< 0.01**	**Yes**	**Yes**	**Yes**
Epirubicin	34.7	39	58.7	< 0.05	Yes	Yes	No
Fludarabine	58.6	62.9	86.2	< 0.05	Yes	No	No
Idarubicin	53.3	57.6	97.7	< 0.01	No	No	No
Irinotecan	30.6	34.9	65.4	< 0.01	Yes	Yes	No
**Mithramycin**	**28.6**	**32.9**	**95**	**< 0.01**	**Yes**	**Yes**	**No**
**Mitoxantrone**	**52.2**	**56.5**	**97.7**	**< 0.01**	**No**	**No**	**No**

**20 μM**							

Cladribine	0	4.1	38.5	< 0.05	No	No	No
Cytarabine	7.9	12.2	32.9	< 0.05	No	No	No
**Gemcitabine**	**28.4**	**32.7**	**70.5**	**< 0.01**	**Yes**	**Yes**	**Yes**
Thioguanine	4.7	9	40.8	< 0.05	No	No	No
**Thiotepa**	**6.2**	**10.5**	**47.2**	**< 0.01**	**No**	**No**	**No**

Values are listed as mean percent viability reduction. Leads employed in an expanded dose response are shown in bold font.

From the TRAIL chemosensitizer leads identified above, five drugs - doxorubicin, gemcitabine, mithramycin, mitoxantrone, and thioTEPA were chosen for additional evaluation. These drugs have been approved by the FDA for chemotherapeutic administration in different malignancies. Doxorubicin and gemcitabine were selected since both have been previously characterized as TRAIL-sensitizing agents in prostate and/or pancreatic cancer cells [21,38-41]. Mithramycin has also been shown to sensitize prostate cancer cells to TRAIL [37] but to our knowledge, the drug has not been previously demonstrated to possess TRAIL sensitization activity in pancreatic cancer cells. Neither mitoxantrone nor thioTEPA has previously been shown to act as TRAIL sensitizers in cancer cells to the best of our knowledge. Additional factors that were used to determine candidates for subsequent characterization included single-agent drug toxicities in the PC3-TR cell line (drugs with lower toxicities were given preference), the total loss of cancer cell viability induced by the combination treatment compared to the single agent treatments, and prior knowledge of the drugs as TRAIL sensitizers.

Additional evaluation involved expanding the range of the drug dose from 0 to 20 μM for doxorubicin and 0 to 100 μM for the other drugs in PC3-TR and PC3 human prostate cancer cells, and the Panc-1 human pancreatic cell cancer line. It is important to note that the screening experiments employed drugs from the JHCCL (frozen 10 mM aliquots), while the lead characterization experiments employed drugs obtained from Sigma (doxorubicin, mitoxantrone) and the NCI/NIH Developmental Therapeutic Program (gemcitabine, mithramycin, thioTEPA) which were reconstituted in either DMSO or water, based on the solvent that was used for the respective drugs supplied in the JHCCL.

Combination treatments were carried out with 10 ng/mL TRAIL, which induced a loss of viability of 4.3% (+/- 3.2%) in PC3-TR cells, 8.4% (+/- 4.2%) in PC3 cells, and 1.4% (+/- 7.4%) in Panc-1 cells, when administered alone. Such low levels of viability loss are demonstrative of the resistance of these cancer cells to TRAIL-induced apoptosis. It is important to note that while PC3-TR cells are derived from PC3 cells, the two lines are inherently different and therefore, it can be expected that the two cell lines respond differently to drug treatments. For example, we have previously shown that closely related prostate cancer cell lines, PC3 and PC3-PSMA cells, demonstrate markedly different behavior in response to nanoparticle treatment [42].

**Figure 3:**
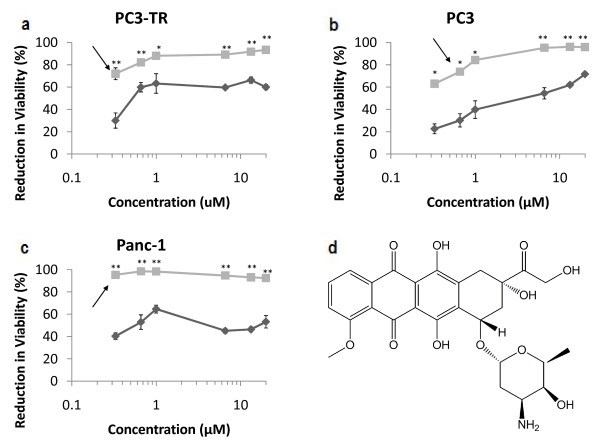
**Dose response for doxorubicin in malignant cell lines**. (a) PC3-TR, (b) PC3 human prostate cancer cells, and (c) Panc-1 pancreatic cancer cells. Single-agent treatments are shown as "dark grey diamonds" and combination treatments with 10 ng/mL of TRAIL are shown as "light grey squares". Asterisks (*) and (**) indicate p-values < 0.05 and < 0.01, respectively. (d) molecular structure of doxorubicin. Arrows indicate the concentrations at which the enhancement in viability reduction is the greatest. Data are presented on a logarithmic scale and the lines connecting data points are for visualization only.

**Figure 4:**
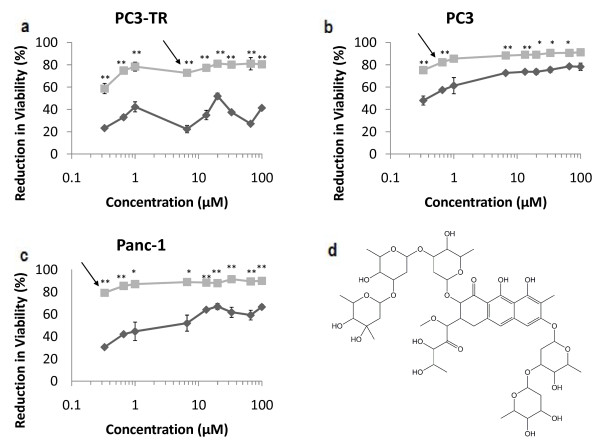
**Dose response for mithramycin (plicamycin) in malignant cell lines**. (a) PC3-TR, (b) PC3 human prostate cancer cells, and (c) Panc-1 pancreatic cancer cells. Single-agent treatments are shown as "dark grey diamonds" and combination treatments with 10 ng/mL of TRAIL are shown as "light grey squares". Asterisks (*) and (**) indicate p-values < 0.05 and < 0.01, respectively. (d) molecular structure of mithramycin. Arrows indicate the concentrations at which the enhancement in viability reduction is the greatest. Data are presented on a logarithmic scale and the lines connecting data points are for visualization only.

**Figure 5:**
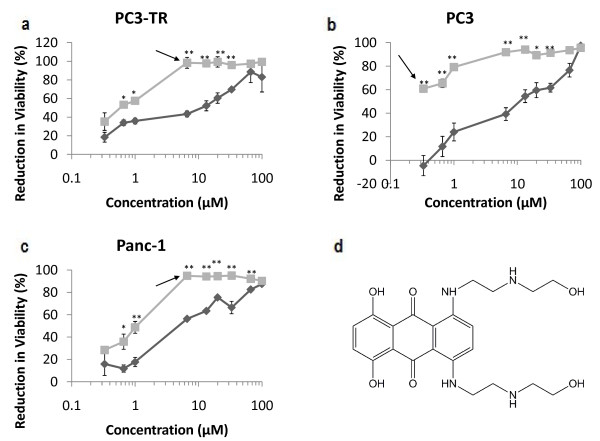
**Dose response for mitoxantrone in malignant cell lines**. (a) PC3-TR, (b) PC3 human prostate cancer cells, and (c) Panc-1 pancreatic cancer cells. Single-agent treatments are shown as "dark grey diamonds" and combination treatments with 10 ng/mL of TRAIL are shown as "light grey squares". Asterisks (*) and (**) indicate p-values < 0.05 and < 0.01, respectively. (d) molecular structure of mitoxantrone. Arrows indicate the concentrations at which the enhancement in viability reduction is the greatest. Data are presented on a logarithmic scale and the lines connecting data points are for visualization only.

The expanded dose range of the lead candidates in the PC3-TR cell line showed trends similar to those seen in the primary screen; all lead candidates induced significant losses in cancer cell viability when used in combination with TRAIL (Figure 3, Figure 4, Figure 5, Additional File 4, Additional File 5). Doxorubicin, mithramycin, and mitoxantrone demonstrated synergistic efficacies in combination with TRAIL in PC3 and Panc-1 cells (Figure 3, Figure 4, Figure 5). Conversely, neither gemcitabine nor thioTEPA demonstrated significant synergy in these cells (Additional File 4, Additional File 5).

The median concentrations of single-agent doxorubicin (Figure 3) that resulted in 50% loss of viability in the cancer cell population compared to the untreated control (LC_50_) were approximately 0.6 μM, 6 μM and 0.6 μM in PC3-TR, PC3, and Panc-1 cells, respectively. However, in combination with 10 ng/mL TRAIL, the LC_50 _values for doxorubicin were 0.25 μM for PC3-TR and PC3 cells, and 0.1 μM (100 nM) for Panc-1 cells, all of which are substantially lower than the single-agent concentrations (Figure 3). The greatest loss of cancer cell viability for the doxorubicin-TRAIL combination treatment compared to single-agent doxorubicin treatment was observed at 0.33 μM (42%), 1 μM (44.4%), and 0.33 μM (55%) for PC3-TR, PC3, and Panc-1 cells, respectively (Figure 3). The cytotoxic effect of doxorubicin is attributed to its DNA intercalation as well as its disruption of cellular functions upon cell membrane binding. Intercalation inhibits nucleotide replication via the stabilization of type II topoisomerase [43]. Doxorubicin is currently approved for the treatment of acute lymphoblastic leukemia, acute myeloblastic leukemia, Wilms' tumor, neuroblastoma, soft tissue and bone sarcomas, breast cancer, ovarian cancer, transitional cell bladder cancer, thyroid cancer, gastric cancer, Hodgkin's disease, malignant lymphoma, bronchogenic cancer, ovarian cancer, AIDS-Related Kaposi's sarcoma, and multiple myeloma. The TRAIL sensitization activities of doxorubicin are due to the ability of the drug to down-regulate the anti-apoptotic protein c-FLIP [38,44], activate pro-apoptotic caspases [45,46] and induce reactive oxygen species (ROS) formation in cancer cells [47]. Thus, the genotoxic activity of doxorubicin activates the internal apoptosis pathway, while simultaneously sensitizing the cell to external, receptor-mediated apoptosis by the TRAIL ligand.

The LC_50 _values for single-agent mithramycin were 20 μM, 0.5 μM and 6 μM for PC3-TR, PC3, and Panc-1 respectively. However, in combination with 10 ng/mL TRAIL, the LC_50 _values for mithramycin were approximately 0.2 μM for PC3-TR and PC3 cells, and 0.1 μM for Panc-1 cells, respectively (Figure 4). Combination treatments with mithramycin (plicamycin) demonstrated the greatest enhancement in loss of cancer cell viability at 6.6 μM (50.5%), 0.66 μM (24.9%) and 0.33 μM (48.6%) in PC3-TR, PC3, and Panc-1 cells respectively, compared to single-agent mithramycin treatment (Figure 4). Importantly, mithramycin demonstrated one of the highest efficacies in combination with TRAIL; a difference of 67% in loss of cancer cell viability was seen for the combination treatment compared to the additive effect of the single-agent treatments (67% increase; Figure 1). Mithramycin is an antineoplastic antibiotic derived from Streptomyces and is approved by the FDA for the treatment of testicular cancer and hypercalcaemia [48]. The antineoplastic properties of mithramycin are likely linked to the binding of mithramycin to GC-rich section of DNA and the subsequent inhibition of RNA synthesis and regulation of transcription [49]. The TRAIL-sensitization activity of mithramycin is not very well characterized, but one study suggests that this activity is caused by the down regulation of X-linked Inhibitor of Apoptosis Protein (XIAP) [37]. XIAP inhibits apoptosis by binding to and inhibiting caspases 3, 7 and 9. Mithramycin is able to prevent the transcription of XIAP through its binding activity to DNA [37,48]. This action would sensitize cancer cells to both, intrinsic and extrinsic apoptosis pathways. However, the extent to which mithramycin activated the intrinsic pathway has not been elucidated.

LC_50 _values for single-agent mitoxantrone were approximately 10 μM for PC3-TR and PC3 cells, and 5 μM for Panc-1 cells. However, in combination with 10 ng/mL TRAIL, the LC_50 _values for mitoxantrone were significantly reduced to 0.6 μM, 0.1 μM and 1 μM for PC3-TR, PC3 and Panc-1 cells, respectively (Figure 5). In the case of mitoxantrone-TRAIL combination treatment, the greatest enhancement in loss of viability was observed at 6.6 μM (55.0%) in PC3-TR, 0.33 μM (60.8%) in PC3, and 6.6 μM (38.5%) in Panc-1 cells (Figure 5), compared to mitoxantrone alone. The chemotherapeutic activity of mitoxantrone is attributed to its ability to intercalate into DNA, resulting in cross-links and strand breaks. Additionally, mitoxantrone also interferes with RNA synthesis and inhibits topoisomerase II [50]. Mitoxantrone has been approved by the FDA for palliative treatment of prostate cancer and curative treatment of acute nonlymphocytic leukemia; the drug has also been recently approved for the treatment of multiple sclerosis. Although we did not find reports that describe the use of mitoxantrone as a TRAIL sensitizer, mitoxantrone has been demonstrated to possess synergistic relationship with Tumor Necrosis Factor (TNF) for inducing apoptosis in cells [51,52]. A detailed evaluation that describes the mechanisms behind the TRAIL-sensitization activity of mitoxantrone is currently under investigation in our laboratory.

In the case of gemcitabine, statistically significant enhancement of viability reduction occurred in PC3-TR at concentrations above 6.6 μM (Additional File 4). Further increase in concentration to 100 μM resulted in an enhancement of cell viability loss up to a maximal value of 29% for the combination treatment over the single drug treatment. Gemcitabine is currently approved for the treatment of ovarian cancer (with carboplatin), breast cancer (with paclitaxel), non-small cell lung cancer (with cisplatin), and pancreatic cancer. Gemcitabine is metabolized intercellularly to active diphosphate and triphosphate nucleosides which work via two mechanisms to inhibit DNA synthesis. First, gemcitabine diphosphate inhibits the enzyme ribonucleotide reductase, which is a catalyst of reactions to form deoxynecleoside triphosphates. Second, gemcitabine triphosphate competes with other deoxynecleoside triphosphates for incorporation into DNA, which is enhanced by the action of gemcitabine diphosphate. Although the TRAIL sensitization activity of gemcitabine is not fully understood, it is hypothesized that the response is related to the activation of pro-apoptotic caspases [26,41]. Previous results have shown that the combination treatment of gemcitabine and TRAIL increases the activation of caspases 8 and 3, while a single agent treatment of gemcitabine increases the activation of only caspase 3 [41]. Although synergy was observed in PC3-TR cells with gemcitabine, we did not see synergy between gemcitabine and TRAIL in PC3 and Panc-1 cells, which differs from other reports in the literature [26,41]. This is likely due to differences in the concentrations of both drug and TRAIL conditions that were employed in these other studies; for example, other studies have employed 100 ng/mL TRAIL, which is ten-fold higher than the concentration used in our study [26,41].

ThioTEPA has been approved by the FDA for the treatment of breast cancer, ovarian cancer, superficial papillary carcinoma of urinary bladder, lymphosarcoma, and Hodgkin's disease. ThioTEPA is a radiomimetic drug that can produce ethylenimine radicals, which disrupt DNA. ThioTEPA showed a 40% difference in viability reduction between the combination treatments and the additive effect of the single agent treatments in the initial screen with PC3-TR; however, only an 8% increase was observed in case of the combination treatment compared to individual treatments in the subsequent experiments (Figure 2 & Additional File 5). This might be due to the different sources of the drug employed in the screening and characterization experiments.

**Figure 6:**
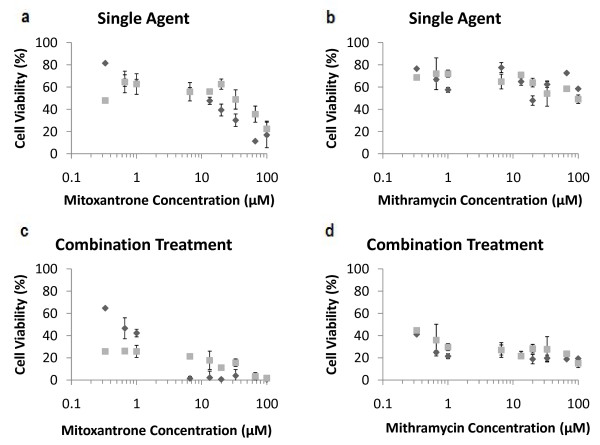
**Evaluation of secondary analysis in PC3-TR cells**. (a) Single agent mitoxantrone, (b) single agent mithramycin, (c) combination treatment with mitoxantrone and 10 ng/mL TRAIL and (d) combination treatment with mithramycin and 10 ng/mL TRAIL. Analysis results performed using the MTT assay are shown as "dark grey diamonds" and analysis results performed using the live/dead stain are shown as "light grey squares". Data are presented on a logarithmic scale.

In order to confirm the above results obtained using the MTT assay, a secondary analysis method using Live/Dead^® ^fluorescence staining was employed to count living cell populations following single agent mitoxantrone/mithramycin treatments as well as TRAIL-based combination treatments in the PC3-TR cell line. Image analysis showed that single agent TRAIL caused a 6% (+/- 4%) decrease in cell viability (not shown). Figure 6 compares cell viability calculated using the MTT assay to the normalized live cell count. The greatest deviation between the MTT and live/dead analysis occurs in the combination mitoxantrone and TRAIL experiments. Deviation is greatest at lower concentrations of mitoxantrone but diminishes as the concentration increases. The average difference between cell viability data for single agent mitoxantrone is 12.9% between the two methods, while the average difference is 15% for the mitoxantrone-TRAIL combinations. The single-agent and combination treatments with mithramycin show excellent agreement between the two cell viability analysis methods. The average difference between analysis methods is 10.4% for mithramycin alone and only 5.6% for the mithramycin-TRAIL combination treatment. These results indicate that the MTT and the live/dead methods are comparable for these systems, which is an indication that the MTT assay is a reliable method for screening and rapidly identifying synergistic combination treatments.

**Figure 7:**
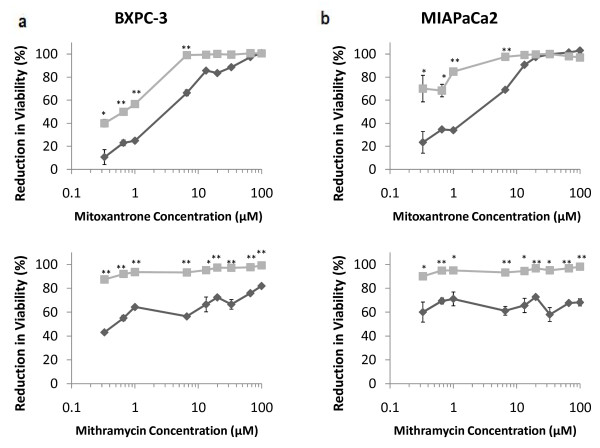
**Evaluation of mitoxantrone and mithramycin in additional malignant lines**. (a) BXPC-3, and (b) MIAPaCa2 human pancreatic cancer cells. Single-agent treatments are shown as "dark grey diamonds" and combination treatments with 10 ng/mL of TRAIL are shown as "light grey squares". Asterisks (*) and (**) indicate p-values < 0.05 and < 0.01, respectively. Data are presented on a logarithmic scale and the lines connecting data points are for visualization only.

Following the identification of mitoxatrone and mithramycin as potent sensitizers of TRAIL-induced apoptosis in cancer cells above, we further evaluated the activities of these drugs in two additional human pancreatic cancer cell lines, BXPC-3 and MIAPaCa2 (Figure 7). Single-agent TRAIL treatments resulted in a loss of viability in 14.3% (+/- 9.8%) in BXPC-3, and 9.2% (+/-3.8%) in MIAPaCa2 cells. The LC_50 _value for single-agent mitoxantrone was approximately 4 μM for BXPC-3 and 3 μM for MIAPaCa2 cells. In comparison, the LC_50 _values for the mitoxantrone-TRAIL combination treatments were 0.66 μM for BXPC-3 and 0.1 μM for MIAPaCa2 cells. Similarly, LC_50 _values for single-agent mithramycin were 0.3 μM for BXPC-3 and 0.2 μM for MIAPaCa2 cells. The LC_50 _values for the mithramycin-TRAIL combination treatments were less than 0.1 μM (100 nM) for both, BXPC-3 and MIAPaCa2 cells. In BXPC-3 cells, the largest difference in loss of viability induced by the drug-TRAIL combination compared to single-agent drug treatment were observed at 6.6 μM (18.5%) and 0.33 μM (42.0%) for mitoxantrone and mithramycin, respectively. In MIAPaCa2 cells, the largest enhancements in viability reduction following combination treatments were observed with mitoxantrone and mithramycin doses of 0.33 μM (37.5%) and 33.3 μM (28.0%), respectively. In case of mitoxantrone, the TRAIL sensitization activity was most effective at lower concentrations of the drug; extensive reduction in cancer cell viability was observed with high concentrations of mitoxatrone in these cells. In contrast, the response was largely invariant as a function of concentration for mithramycin in these cells. These results are similar to those observed with other cancer cell lines (Figure 4, Figure 5). Taken together, the significant reduction in the LC_50 _values observed in case of combination treatments, compared to the additive effects of single-agent treatments of chemotherapeutic drugs and TRAIL, further demonstrates the efficacy this approach for the ablation of pancreatic cancer cells.

### The Combination Treatment of Low-dose Mitoxantrone and TRAIL is Selective towards Malignant Pancreatic Cells Compared to Normal Pancreatic Epithelial Cells

With the exception of gemcitabine and thioTEPA, the largest enhancements in viability reduction occurred at sub-micromolar or low micromolar concentrations for the other drugs. This is significant since the use of lower concentrations of these genotoxins can reduce damage to healthy tissue during therapy. This was demonstrated further by comparing single-agent verses the combination treatment LC_50 _values for each of the chemotherapeutic drugs. In the case of doxorubicin, mithramycin and mitoxantrone, the LC_50 _values decreased when each of these drugs was used in combination with TRAIL regardless of the cell line. With doxorubicin, this decrease in the LC_50 _value was relatively small in PC3-TR (0.6 μM to 0.25 μM) and Panc-1 (0.6 μM to 0.1 μM) cells, but significant in PC3 cells (6 μM to 0.25 μM). Mithramycin exhibited a relatively small change in the LC_50 _value for PC3 cells (0.5 μM to 0.2 μM), a moderate change for Panc-1 cells (6 μM to 0.1 μM), and the largest total change for PC3-TR cells (20 μM to 0.2 μM). Mitoxantrone demonstrated a moderate decrease in the LC_50 _value for Panc-1 cells (5 μM to 1 μM) and significantly larger decreases in LC_50 _values for the drug in combination with TRAIL in both, PC3-TR (10 μM to 0.6 μM) and PC3 (10 μM to 0.1 μM) cells.

**Figure 8:**
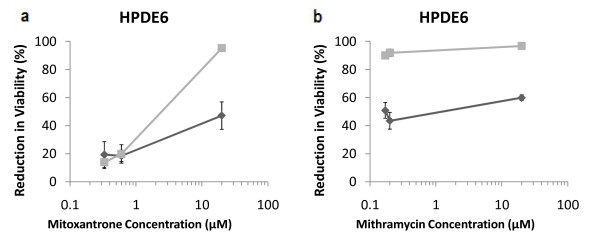
**Evaluation of treatments in non-malignant cell line**. Mitoxantrone (a) and mithramycin (b) in HPDE6 human pancreatic duct epithelial cells. Single-agent treatments are shown as "dark grey diamonds" and combination treatments with 10 ng/mL of TRAIL are shown as "light grey squares". Data are presented on a logarithmic scale and the lines connecting data points are for visualization only.

We tested the effects of mitoxantrone and mithramycin in the non-malignant HPDE6 [53,54] pancreatic cells in order to determine the selectivity of these two drugs and their combination with TRAIL for malignant cells compared to normal cells (Figure 8). Single agent TRAIL showed a viability loss of 3.3% (+/- 12.4%) in HDPE6 cells compared to untreated cell control, suggesting that TRAIL shows little to no activity in these cells. Low concentrations (0.33 and 0.6 μM) of single-agent mitoxantrone also exhibited minimal loss in HPDE6 cell viability, while the 20 μM treatment resulted in a loss of viability of approximately 50% cells. Single agent mithramycin treatment, however, resulted in a loss of viability in 40%-60% of the cell population even at the lower concentrations, which was considerably higher than that observed with mitoxantrone (Figure 8). A comparison of the single agent treatments and the combination treatments for mitoxantrone indicated selectivity of the drug at lower concentrations. In HDPE cells, 0.33 and 0.6 μM mitoxantrone in combination with 10 ng/mL TRAIL induced a 13.9% (+/- 4.5%) and 19.8% (+/-6.6%) loss of viability respectively, while mitoxantrone alone resulted in 19.3% (+/- 9.2%) and 18.4% (4.1%) at these doses. Importantly, single agent mitoxantrone and mitoxantrone + TRAIL induced a respective loss of cell viability of 16% and 28.4% in Panc-1 cells, 10.6% and 40% in BXPC3 cells, 23.4% and 70% in MIAPaCa2 (0.33 μM mitoxantrone). These conditions indicate that the combination of low-dose mitoxantrone with TRAIL is selective towards malignant pancreatic cells compared to normal cells. As may be expected, this selectivity is lost at higher concentrations of mitoxantrone. We did not observe significant selectivity for cancer cells compared to non-malignant cells in case of mithramycin. While the results with mitoxantrone are promising due to the observed selectivity, an effective chemotherapeutic window of operation may be available for the mithramycin-TRAIL combination in vivo.

### Higher Concentrations of TRAIL in Combination Treatments do not Demonstrate Increased Efficacies

**Figure 9:**
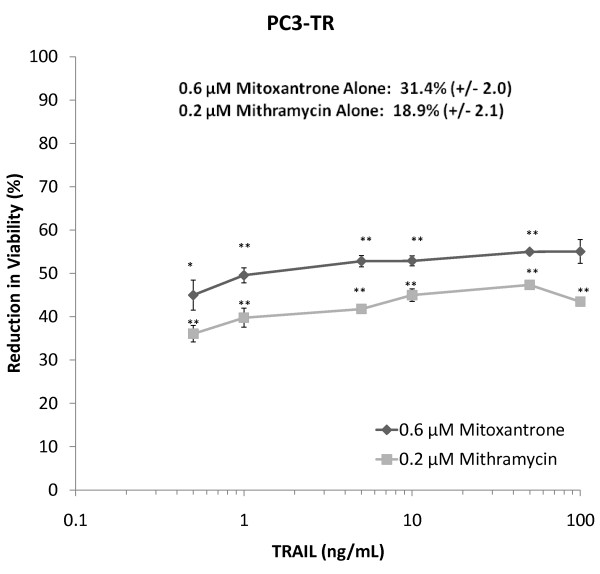
**Dose response of TRAIL with single mitoxantrone and mithramycin concentrations in PC3-TR cells**. Mitoxantrone treatments are shown as "dark grey diamonds" and mithramycin treatments are shown as "light grey squares". Asterisks (*) and (**) indicate p-values < 0.05 and < 0.01, respectively. Data are presented on a logarithmic scale and the lines connecting data points are for visualization only.

The previous studies were carried out with different concentrations of the chemosensitizer drug followed by a single dose of TRAIL (Additional File 1). Following identification of mitoxantrone and mithramycin as potential TRAIL sensitizers, we investigated the effect of different TRAIL concentrations in combination with a single dose of these sensitizer drugs. LC_50 _values for mitoxantrone-TRAIL and mithramycin-TRAIL combination treatments were 0.6 and 0.2 μM, respectively, for PC3-TR cells. The single-agent 0.6 μM mitoxantrone treatment, resulted in a loss of viability of 31.4% (+/- 2.0%) in PC3-TR cells compared to the untreated control (Figure 9), which is in good agreement with the data trend shown in Figure 5. The single agent treatment of 0.2 μM mithramycin resulted in a loss of viability of 18.9% (+/- 2.1%) of cells compared to untreated cells (Figure 9), which is also in good agreement with data shown in Figure 4. As expected, the loss of cancer cell viability upon treatment with single-agent 0.6 μM mitoxantrone and 0.2 μM mithramycin was less than 50%, since these are less efficacious than the combination treatments. These single-agent concentrations were chosen to investigate the efficacy of different TRAIL doses, which were varied from 0-100 ng/mL (Figure 9). Over the concentration range studied for TRAIL, the overall reduction in cell viability between the lowest (0.5 ng/mL) and highest (100 ng/mL) TRAIL concentrations increases by only 10% for both drug treatments, indicating that lower TRAIL doses (e.g. 10 ng/mL) may possess sufficient activity in the current combination treatments.

### Sequential vs. Simultaneous Combination Treatments

**Figure 10:**
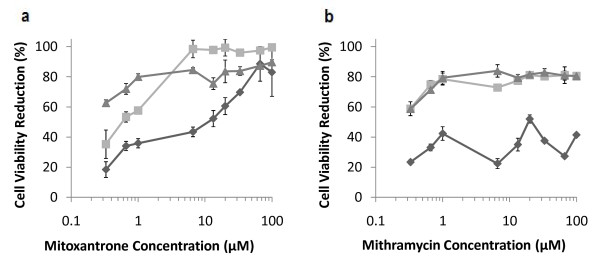
**Comparison between different treatment methodologies in PC3-TR cells**. (a) Mitoxantrone (b) and mithramycin in PC3-TR prostate cancer cells. Single-agent treatments are shown as "dark grey diamonds", sequential combination treatments with 10 ng/mL of TRAIL are shown as "light grey squares" and simultaneous combination treatments with 10 ng/mL of TRAIL are shown as "grey triangles". Data are presented on a logarithmic scale and the lines connecting data points are for visualization only.

In the previous experiments, combination treatments were performed in a sequential format by first treating cells with a sensitizing drug for 24 hours, removing the drug from the cells, and then applying a TRAIL treatment for an additional 24 hours. An alternate combination treatment methodology is to apply both the sensitizing drug and TRAIL "simultaneously" in which both, the drug and TRAIL are administered together. In our hands, simultaneous treatments with mitoxantrone demonstrated a higher loss of PC3-TR cell viability for low concentrations of the drug compared to the sequential treatments (Figure 10). However, this trend is reversed at higher drug concentrations.

The overall increase in loss of cancer cell viability between the lowest concentration of mitoxantrone (0.33 μM) and the highest concentration of mitoxantrone (100 μM) for the simultaneous treatments is about 25% (Figure 10), compared to 65% for sequential treatments. Interestingly, mithramycin did not show large differences in the loss of cell viability between the sequential and the simultaneous combination treatments. The difference between the two drugs and their dependence on treatment order is likely most closely related to the kinetics of how each drug is processed and how quickly the sensitization effect is achieved. In the case of mithramycin it is likely that the kinetics of sensitization are rapid and that even when the drugs are co-administered, there is sufficient time for the drug to sensitize the cells to TRAIL mediated apoptosis. On the other hand, it is possible that the kinetics of sensitization are slower for mitoxantrone and that the synergy between mitoxantrone and TRAIL is highest when the drug has sufficient time to overcome cellular resistances to TRAIL. We are currently following up on these observations in our laboratory.

### Mitoxantrone-TRAIL and Mithramycin-TRAIL Combinations induce Apoptosis in PC3-TR Cells

**Figure 11:**
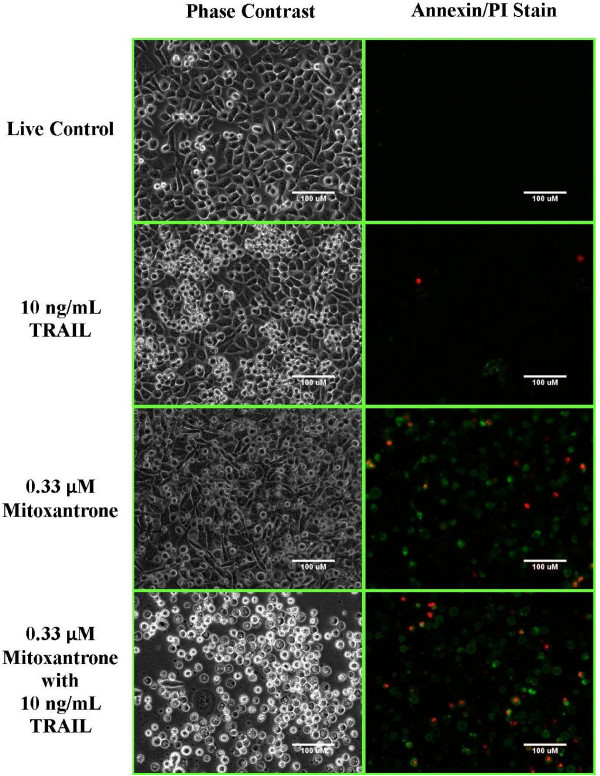
**Microscopy images of mitoxantrone treated PC3-TR cells**. Left panels are phase contrast images and right panels are fluorescence images visualized with an annexin V and propidium iodide stain. Apoptotic cells exhibit green fluorescence only. Dead cells can exhibit either lone red fluorescence or red and green fluorescence simultaneously. Live cells are non-fluorescent. Arrows identify apoptotic cells.

In order to verify the induction of apoptosis, cells were treated with an annexin V and propidium iodide (PI) stain following incubation with single-agent and combination drug treatment. The annexin V/PI stain distinguishes between apoptotic and necrotic cells based on fluorescence. In this assay, live cells will not fluoresce, early apoptotic cells fluoresce green, while late apoptotic and necrotic cells can fluoresce either red or demonstrate both red and green fluorescence. Images were acquired for single agent and combination treatments of mitoxantrone (Figure 11) and mithramycin (Additional File 6) in PC3-TR cells. As expected, the live cell control and the 10 ng/mL TRAIL-alone treatment control demonstrated little to no fluorescence. Single agent treatment with mitoxantrone (0.33 μM) resulted in both apoptotic and necrotic cell populations, although the majority of the cell population consisted of live cells. The corresponding mitoxantrone-TRAIL simultaneous combination treatment results in a large population of apoptotic and necrotic cells (Figure 11), which also demonstrate changes in cell. Mithramycin treatments demonstrated similar trends and showed a clear decrease in cell population between single agent and combination treatments. This validates results shown in Figure 4. Overall, these results indicate that TRAIL-based combination treatments with mitoxantrone and mithramycin induce apoptosis in PC3-TR cells.

## Conclusions

In the current study, fifty-five FDA- and foreign-approved antineoplastic drugs were screened in order to identify chemotherapeutic candidates that sensitized malignant prostate and pancreatic cells to TRAIL-induced apoptosis. The initial screen was performed using a TRAIL resistant prostate cancer cell line (PC3-TR) and several drugs were identified as potential sensitizing agents. The screen was able to identify drugs with previously unknown TRAIL sensitization activities in prostate as well as pancreatic cancer cells, which can lead to the identification of new chemotherapeutic drug combinations and therefore potentially increase therapeutic options against these malignancies. Future work will involve expansion of the screen to other drug candidates, a detailed investigation into the mechanisms responsible for the sensitization activities of the leads and an evaluation of their efficacy in relevant animal models of prostate and pancreatic tumors.

## Competing interests

The authors declare that they have no competing interests.

## Authors' contributions

KR, AJ, and DT designed the experiments, DT and CEP carried out the experimental work, HH carried out the experimental work with HDPE cells. KR and DT wrote and edited the manuscript; AJ and HH read and edited the manuscript. All authors have read and approved the final manuscript.

## Pre-publication history

The pre-publication history for this paper can be accessed here:

http://www.biomedcentral.com/1471-2407/11/470/prepub

## Supplementary Material

Additional file 1 **Dose response for TRAIL as a single agent treatment in the PC3-TR cell line**. Cells were incubated with TRAIL for a 24-hour period followed by analysis with MTT. TRAIL at 10 ng/mL concentration causes a 4.3% (+/- 3.2%) increase in viability reduction compared to the live control. Click here for file

Additional file 2 **Flow chart outlining the screening process of the 55 drugs tested**. Click here for file

Additional file 3 **Single-agent drug toxicity pre-screen (20 μM) with PC3-TR cell line**. Drugs found to induce a decrease in cell viability greater than 30% were retested at an alternate drug concentration of 10 μM (drugs circled). Drugs were incubated with the cells for a 24-hour period after which an MTT analysis was performed. Click here for file

Additional file 4 **Dose response for gemcitabine in malignant cell lines**. (a) PC3-TR, (b) PC3 human prostate cancer cells, and (c) Panc-1 pancreatic cancer cells. Single-agent treatments are shown as "dark grey diamonds" and combination treatments with 10 ng/mL of TRAIL are shown as "light grey squares". Asterisks (*) and (**) indicate p-values < 0.05 and < 0.01, respectively. (d) molecular structure of gemcitabine. Arrows indicate the concentrations at which the enhancement in viability reduction is the greatest. Data are presented on a logarithmic scale and the lines connecting data points are for visualization only. Click here for file

Additional file 5 **Dose response for thioTEPA in malignant cell lines**. (a) PC3-TR, (b) PC3 human prostate cancer cells, and (c) Panc-1 pancreatic cancer cells. Single-agent treatments are shown as "dark grey diamonds" and combination treatments with 10 ng/mL of TRAIL are shown as "light grey squares". Asterisks (*) and (**) indicate p-values < 0.05 and < 0.01, respectively. (d) molecular structure of thioTEPA. Data are presented on a logarithmic scale and the lines connecting data points are for visualization only. Click here for file

Additional file 6 **Microscopy images of mithramycin treated PC3-TR cells**. Left panels are phase contrast images and right panels are fluorescence images visualized with an annexin V and propidium iodide stain. Apoptotic cells exhibit green fluorescence only. Dead cells can exhibit either lone red fluorescence or red and green fluorescence simultaneously. Live cells are non-fluorescent. Arrows identify apoptotic cells. Click here for file
