# Supplementary figures and images for: Parallel screening of FDA-approved antineoplastic drugs for identifying sensitizers of TRAIL-induced apoptosis in cancer cells
Source: BMC Cancer. 2011 Nov 1;11:470. doi: 10.1186/1471-2407-11-470 (PMC3223153; doi:10.1186/1471-2407-11-470)

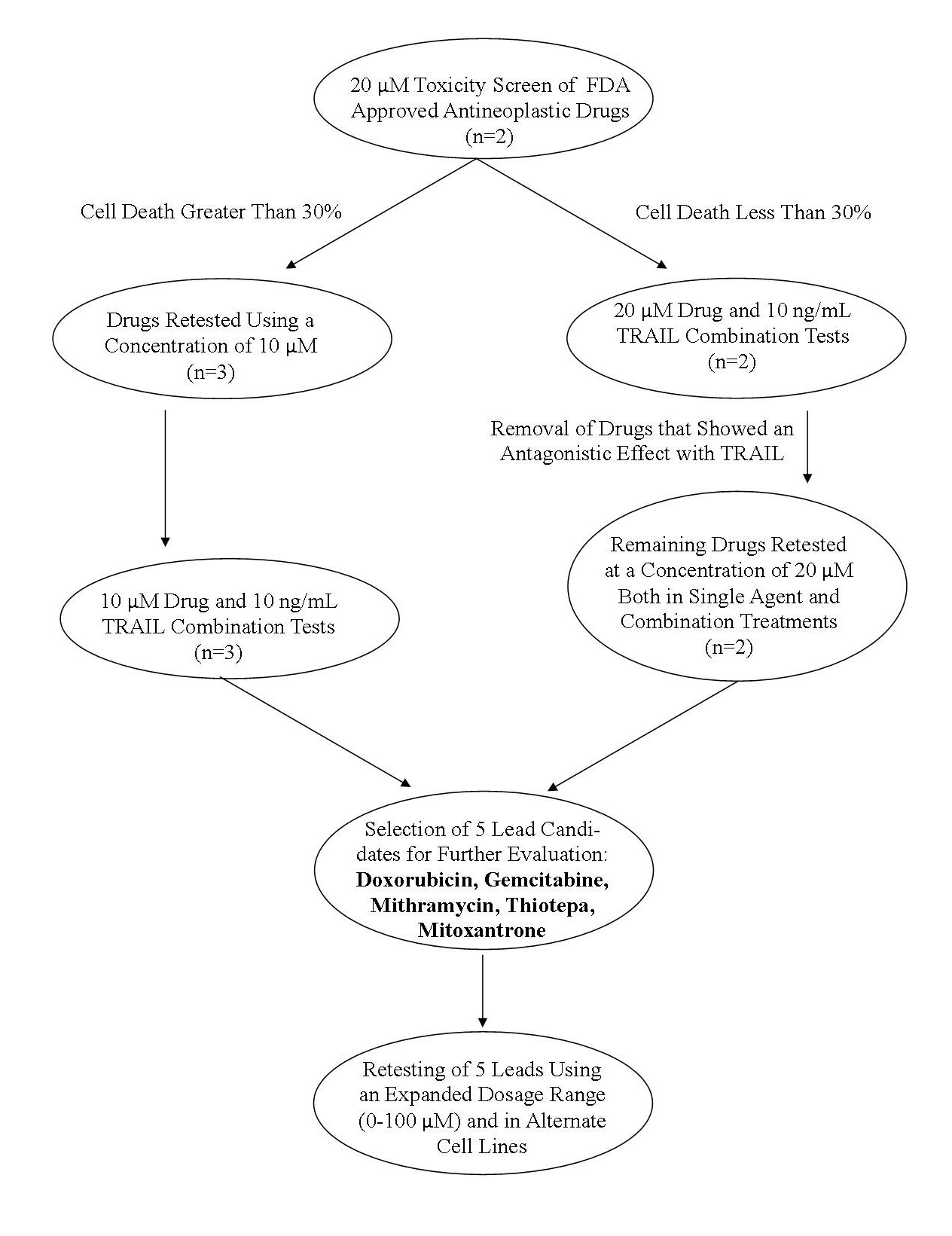

Supplement: Additional file 2 — Flow chart outlining the screening process of the 55 drugs tested. [file 1471-2407-11-470-S2.DOC]

**
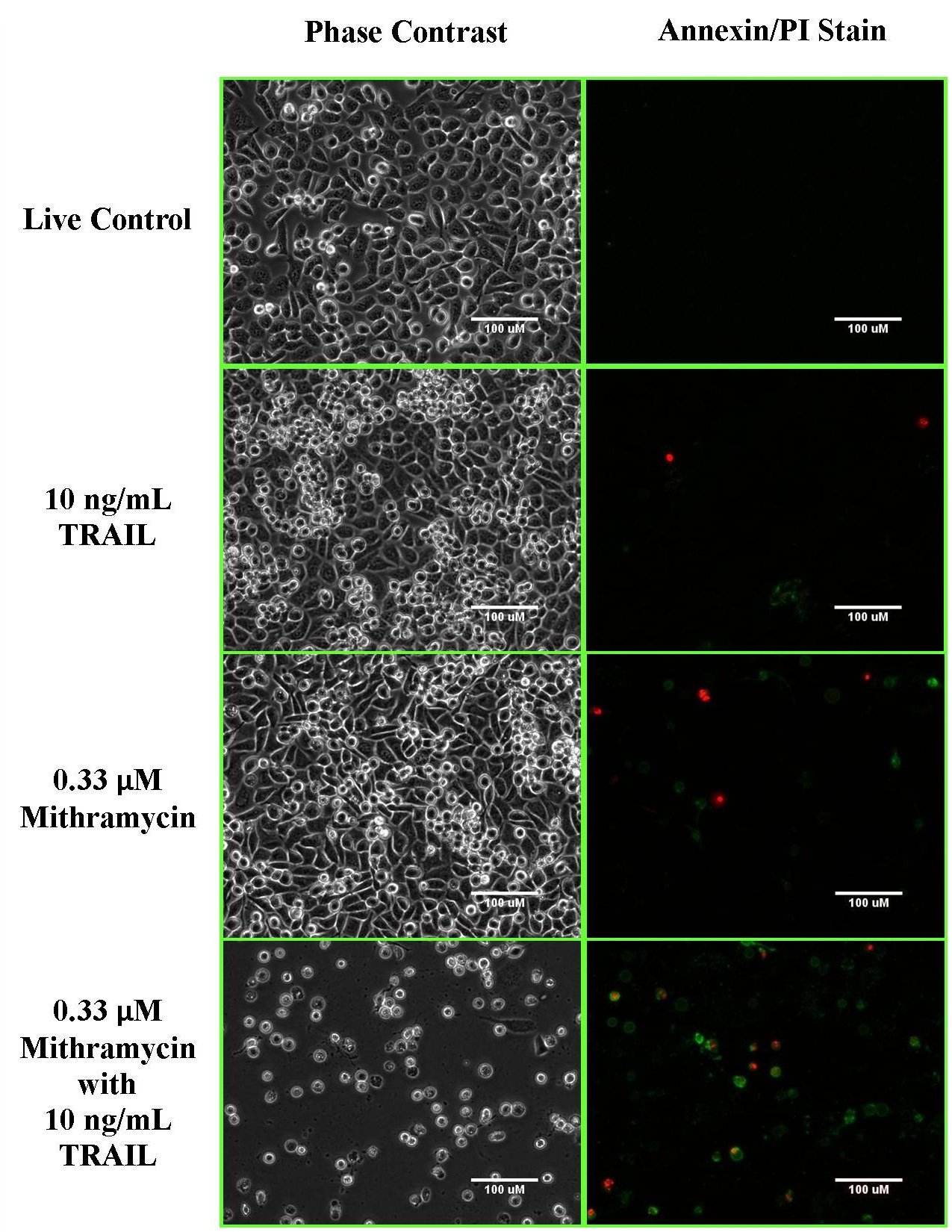
**

Supplement: Additional file 6 — Microscopy images of mithramycin treated PC3-TR cells. Left panels are phase contrast images and right panels are fluorescence images visualized with an annexin V and propidium iodide stain. Apoptotic cells exhibit green fluorescence only. Dead cells can exhibit either lone red fluorescence or red and green fluorescence simultaneously. Live cells are non-fluorescent. Arrows identify apoptotic cells. [file 1471-2407-11-470-S6.DOC]
