# Supplementary material for: Parallel screening of FDA-approved antineoplastic drugs for identifying sensitizers of TRAIL-induced apoptosis in cancer cells
Source: BMC Cancer. 2011 Nov 1;11:470. doi: 10.1186/1471-2407-11-470 (PMC3223153; doi:10.1186/1471-2407-11-470)
Supplement: Supplementary file 2 [file 1471-2407-11-470-S1.DOC]

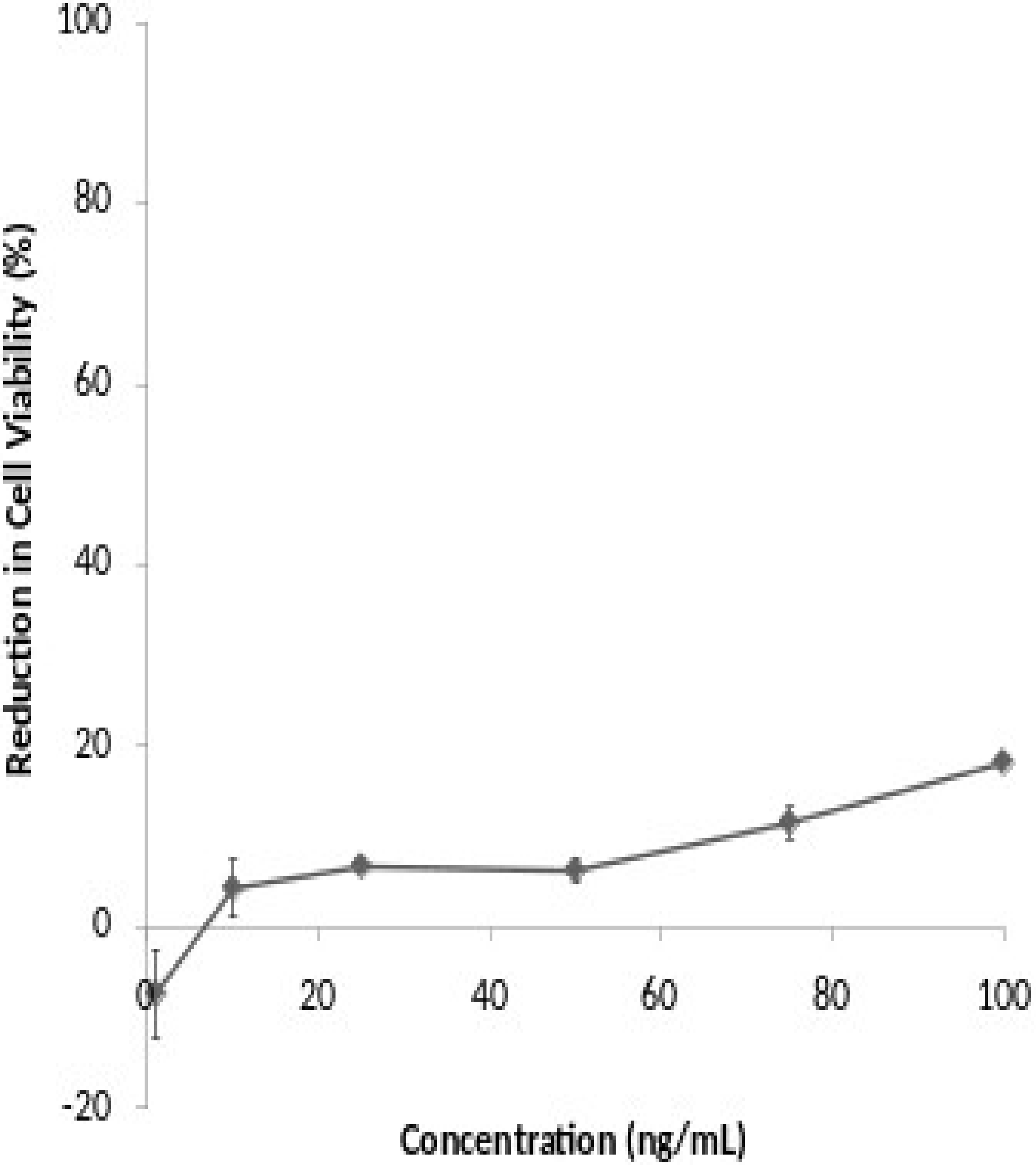

Reduction in Cell Viability (%)
100
80
60
40
20
0
-20
0
20
40
60
80
100
Concentration (ng/mL)
