# Supplementary material for: Parallel screening of FDA-approved antineoplastic drugs for identifying sensitizers of TRAIL-induced apoptosis in cancer cells
Source: BMC Cancer. 2011 Nov 1;11:470. doi: 10.1186/1471-2407-11-470 (PMC3223153; doi:10.1186/1471-2407-11-470)
Supplement: Supplementary file 3 [file 1471-2407-11-470-S3.DOC]

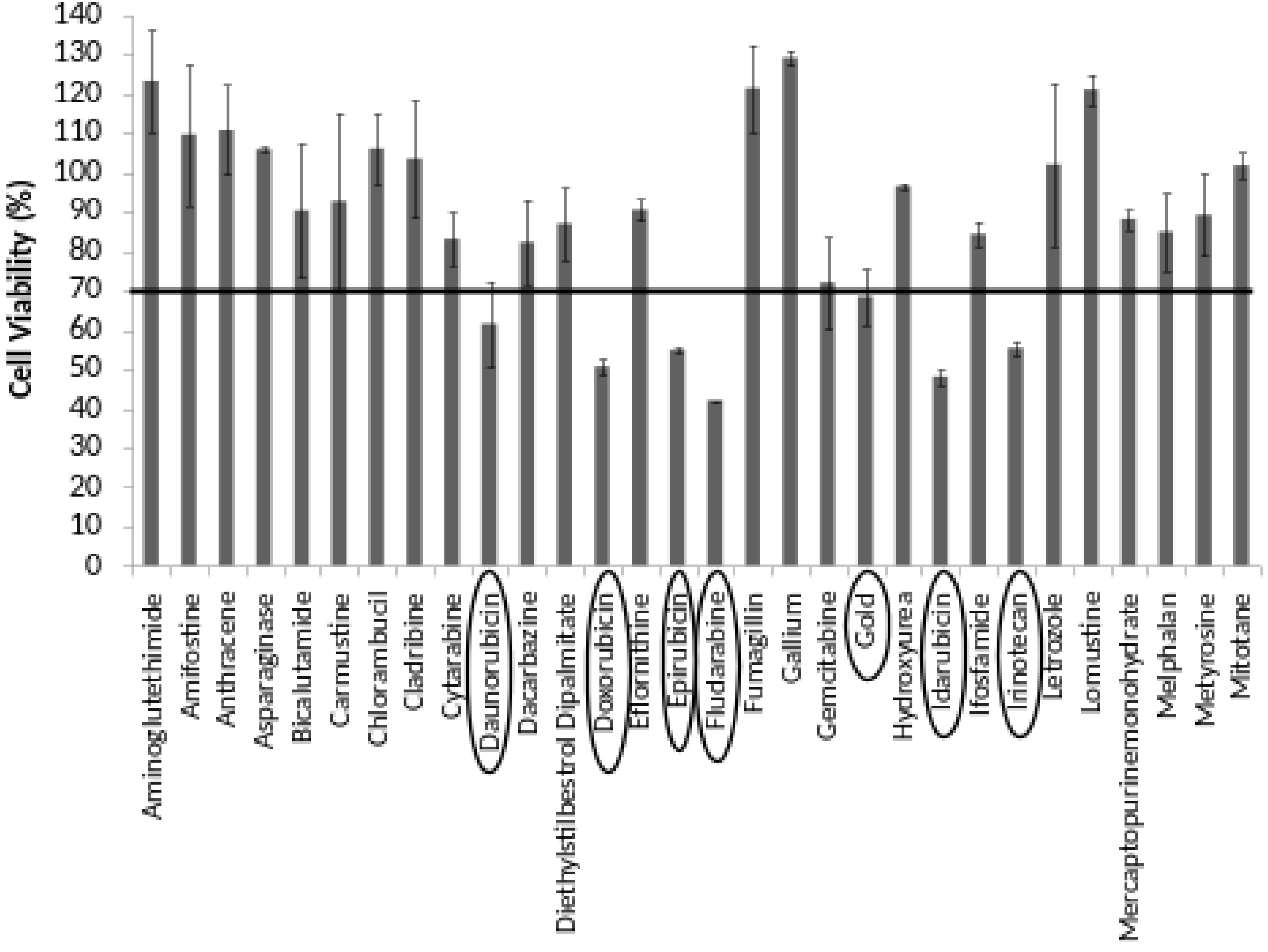
Cell Viability (%)
0
10
20
30
40
50
60
70
80
90
100
110
120
130
140
Aminoglutethimide
Amifostine
Anthracene
Asparaginase
Bicalutamide
Carmustine
Chlorambucil
Cladribine
Cytarabine
Daunorubicin
Dacarbazine
Diethylstilbestrol Dipalmitate
Doxorubicin
Eflornithine
Epirubicin
Fludarabine
Fumagillin
Gallium
Gemcitabine
Gold
Hydroxyurea
Idarubicin
Ifosfamide
Irinotecan
Letrozole
Lomustine
Mercaptopurinemonohydrate
Melphalan
Metyrosine
Mitotane
