# Supplementary material for: Parallel screening of FDA-approved antineoplastic drugs for identifying sensitizers of TRAIL-induced apoptosis in cancer cells
Source: BMC Cancer. 2011 Nov 1;11:470. doi: 10.1186/1471-2407-11-470 (PMC3223153; doi:10.1186/1471-2407-11-470)
Supplement: Supplementary file 4 [file 1471-2407-11-470-S4.DOC]

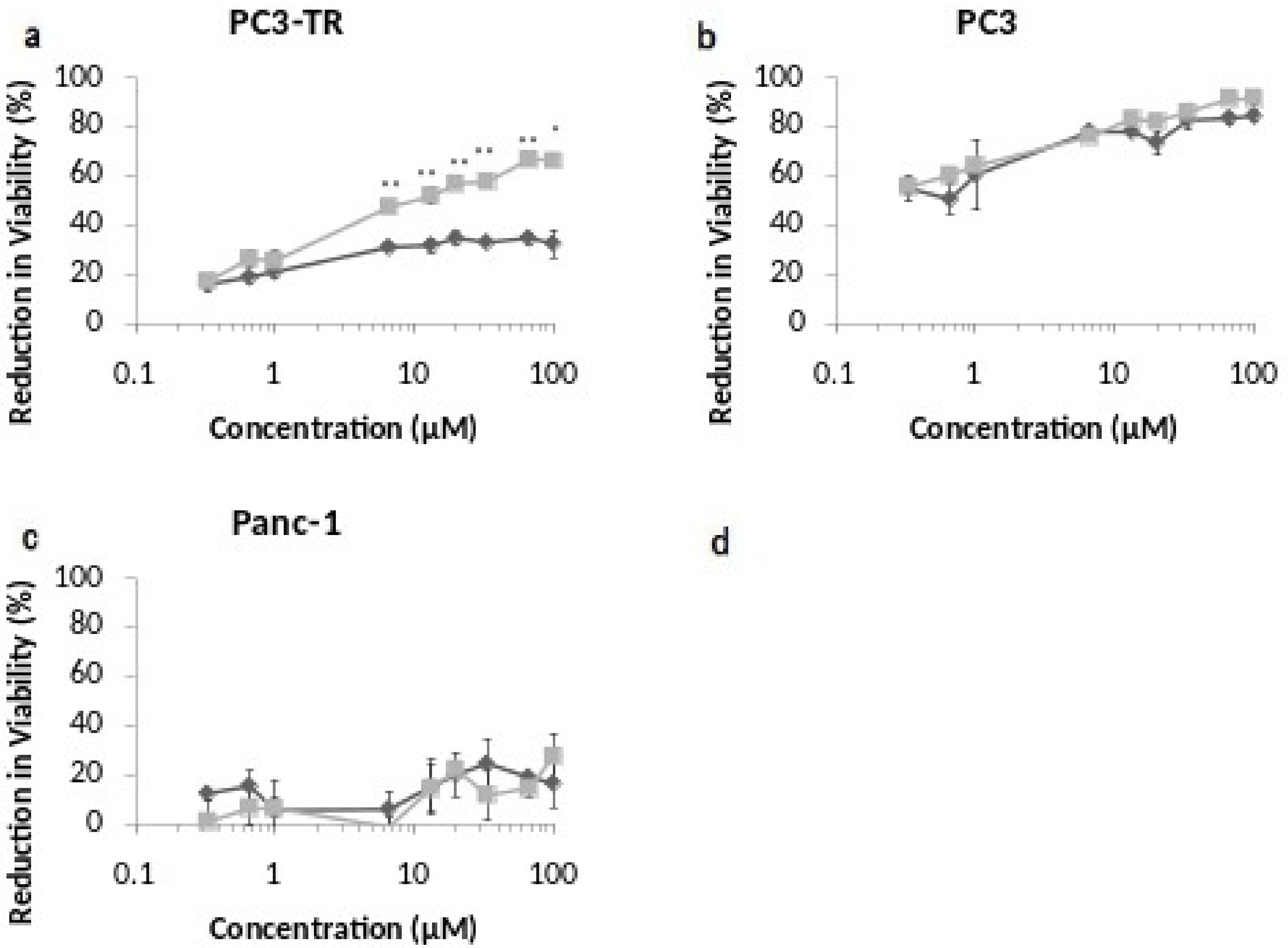
a
PC3-TR
b
PC3
c
Panc-1
d
Reduction in Viability (%)
Concentration (µM)
0
20
40
60
80
100
0.1
1
10
100
