# Supplementary material for: Parallel screening of FDA-approved antineoplastic drugs for identifying sensitizers of TRAIL-induced apoptosis in cancer cells
Source: BMC Cancer. 2011 Nov 1;11:470. doi: 10.1186/1471-2407-11-470 (PMC3223153; doi:10.1186/1471-2407-11-470)
Supplement: Supplementary file 5 [file 1471-2407-11-470-S5.DOC]

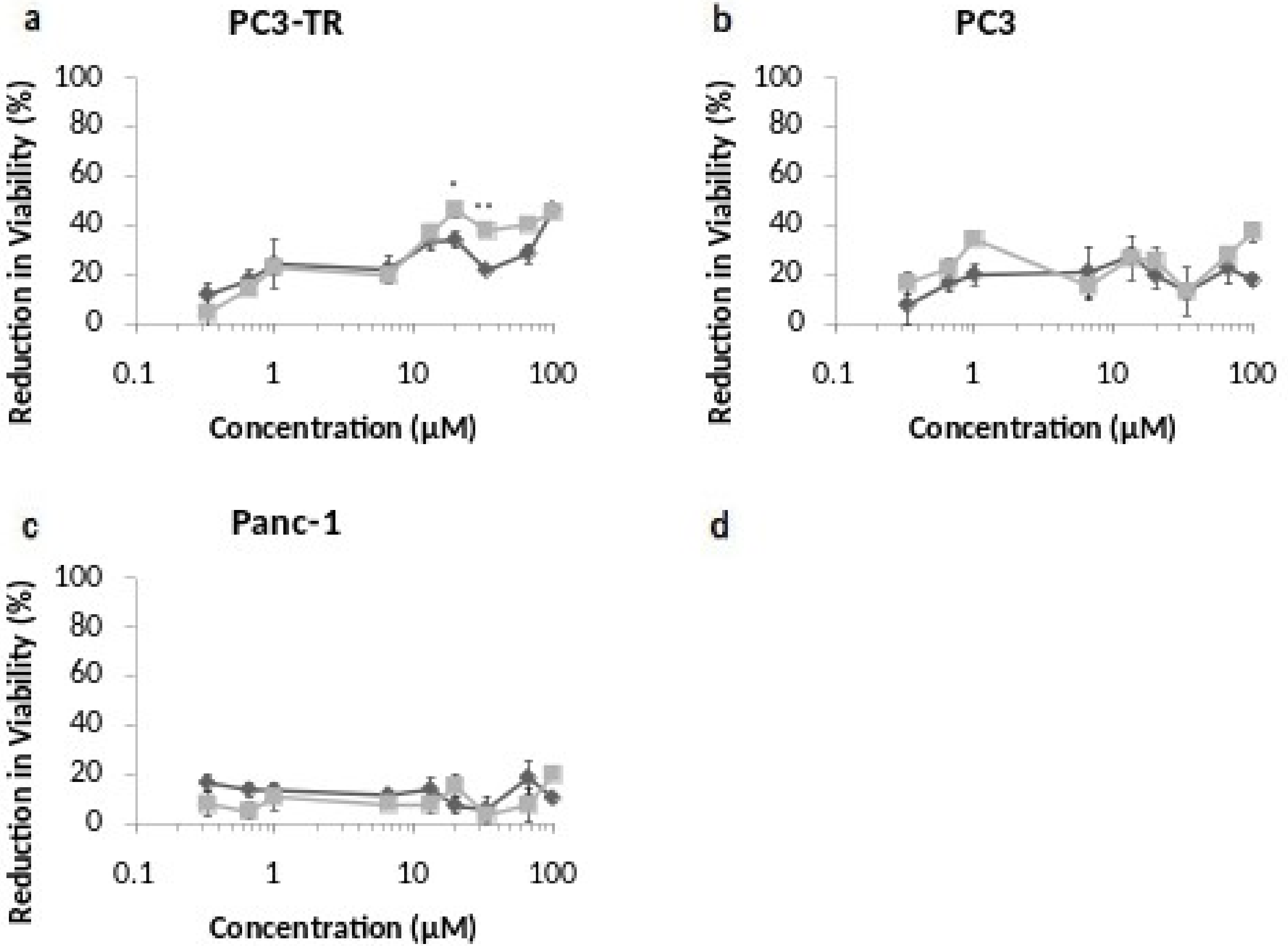
a
PC3-TR
Reduction in Viability (%)
100
80
60
40
20
0
0.1
1
10
100
Concentration (µM)
b
PC3
Reduction in Viability (%)
100
80
60
40
20
0
0.1
1
10
100
Concentration (µM)
c
Panc-1
Reduction in Viability (%)
100
80
60
40
20
0
0.1
1
10
100
Concentration (µM)
d
